# Developmental Enamel Defects (DDE) and Their Association with Oral Health, Preventive Procedures, and Children’s Psychosocial Attitudes towards Home Oral Hygiene: A Cross-Sectional Study

**DOI:** 10.3390/ijerph17114025

**Published:** 2020-06-05

**Authors:** Alessandro Nota, Luca Palumbo, Giuseppe Pantaleo, Enrico Felice Gherlone, Simona Tecco

**Affiliations:** 1 Dental School, Vita-Salute San Raffaele University and Istituto di Ricovero e Cura a Carattere Scientifico San Raffaele, 20132 Milan, Italy; palumbo.niro@hotmail.it (L.P.); gherlone.enrico@hsr.it (E.F.G.); tecco.simona@hsr.it (S.T.); 2 UniSR-Social.Lab, Faculty of Psychology, Vita-Salute San Raffaele University, 20132 Milan, Italy; pantaleo.giuseppe@hsr.it

**Keywords:** developmental enamel defects, caries, dental, oral health, psychosocial attitudes and habits, behaviors

## Abstract

Background: Developmental enamel defects (DDE) exert significant effects both on esthetics and occlusal function and prevention should be the general clinical approach to DDE. The purpose of this cross-sectional study was, therefore, to detect, within a pediatric sample, any significant association between DDE and children’s psychosocial attitudes towards home oral hygiene, as well as potential associations between primary preventive procedures and DDE. Methods: 394 schoolchildren (197 males and 197 females, 8.9 ± 1.4 years) underwent an intra-oral examination; they were then interviewed with a brief questionnaire. Results: 5–6% and 12–14% of participants had, respectively at least one molar and one incisor affected by DDE. In general, no associations were observed in the examined clinical categories (caries and other oral health indices). A strong relationship was found between the presence of molar DDE and children’s positive vs. negative psychosocial attitudes towards home oral hygiene. Conclusions: The probability of finding DDE in our sample of examined children was approximately more than doubled for children with negative psychosocial attitudes towards home oral hygiene, than for children with positive attitudes towards home oral hygiene.

## 1. Introduction

Enamel formation or amelogenesis is a genetic mechanism prone to environmental disturbances bringing to the so-called enamel defects that, if produced during tooth development, cannot undergo further repair. For this reason, every individual’s enamel is a record of the first 8 or 9 years of their life, when the crowns are formed [1,2]. Enamel defects are known to occur due to a depressed activity of the enamel-forming ameloblasts, which in turn results in the formation of linearly distributed pits or grooves.

Developmental defects of enamel (DDE) can be classified into three types: demarcated opacity, diffuse opacity and hypoplasia. Opacities, which alter the translucency of enamel, are also called hypomineralization defects. Hypoplasia is the result of imbalance during the enamel matrix formation (secretion phase), whereas hypomineralization is the result of imbalance during the enamel mineralization (maturation phase). Hypoplasia is a quantitative defect associated with a reduced thickness of enamel. It can be considered as an external defect that can be present as shallow or deep fossae with horizontal or vertical grooves and anomaly in the tissue translucency. A white or yellowish/brownish area can be observed, and there is no alteration of the thickness [3]. These DDE can exert significant effects on esthetics, tooth sensitivity and occlusal function [4,5,6]. Moreover, enamel hypoplasia has been described as one of the predisposing factors for early childhood caries (ECC) and erosion [7,8]. Thus, primary dentition with incomplete enamel calcification on pits and fissures provides a suitable site for the adhesion and colonization of cariogenic bacteria [6]. Consequently, ECC will develop more rapidly on altered tooth surfaces [7].

In addition, restorative procedures for the altered enamel structure in teeth with DDE often create difficulties with anesthetizing the tooth and bonding of the restoration to the enamel [8]. Thus, prevention should still be the general clinical approach to DDE [9,10].

From a clinical point of view, it is apparent that preventive procedures (such as sealings, professional fluoride applications, and adequate home oral hygiene) have better outcomes when patients—especially children—hold a general positive attitude towards those procedures, particularly towards home oral hygiene. Recent studies showed some faults in the motivation, awareness and general knowledge about oral health and prevention in preschool children and their parents [11,12,13], while hospital or school based oral health programs seem to be able to significantly improve children’s level of oral hygiene [14,15].

Therefore, in-line with the data of previous research findings focusing on both clinical and psychological indexes of patients’ attitudes towards oral hygiene [16], clinicians should examine the views and habits that their patients hold towards home oral hygiene and preventive procedures. This analysis is particularly important for children affected by DDE, because of the implications this condition has in smile esthetics and occlusal function.

In-depth knowledge of these aspects could provide useful information on how the clinician should approach, from a clinical and psychosocial point of view, a child with DDE. The gathering of information on both clinical and psychosocial indices related to patient’ physical and psychological health is becoming increasingly decisive in various medical disciplines [17].

The psychosocial attitude that a child holds towards home oral hygiene can play a fundamental role in the clinical management of children in this broad area of intervention. Primary prevention appears to be the most appropriate strategy in the management of DDE, but successful prevention strategies and protocols can only be effective when patients hold substantially positive psychological attitudes towards the enactment of such procedures.

Today, the literature lacks data in this specific area of investigation. More specifically, we would need at least preliminary, but reliable data on a possible association between DDE and patients’ psychosocial attitudes toward home oral hygiene (positive vs. negative). The diffusion of preventive dentistry among young patients affected by DDE should be explored well.

Therefore, the purpose of the present cross-sectional study was to examine the prevalence of DDE in a pediatric sample, and then to detect any plausible associations: (a) between DDE and tooth decay, aphtae or other oral health and preventive dentistry variables, and (b) between DDE and children’s key psychosocial (positive vs. negative) attitudes towards home oral hygiene.

## 2. Materials and Methods

This observational study aimed at investigating the prevalence of molar and incisor DDE in a pediatric Italian sample, together with possible associations of DDE with tooth decay, aphthae and other variables related with oral health and preventive dentistry procedures and with children’s (positive vs. negative) psychosocial attitudes towards home oral hygiene.

The sample included 394 schoolchildren (197 males and 197 females, mean age 8.9 ± 1.4 years) from Milan, Italy. Data were collected during a prevention project on celiac disease, implemented through an agreement between the City Administration of Segrate (Milan, Italy) and the Vita-Salute San Raffaele University (Milan, Italy). At the outset of the study, all students in the School were considered as potentially eligible for participation in the study. The study protocol received the ethical approval by the Institutional Review Board of the Vita-Salute San Raffaele University (Project “Il cavo orale. Un’attenta sentinella”, November 2014). This study adheres to STROBE guidelines for reporting observational research [18]. The STROBE checklist was strictly followed to report the main findings of the study. The procedures of this study follow those described in a previous report [9].

The data were collected between 15 November 2014 and 21 January 2015. A thorough illustration of the whole project can be found in a previous report [9]. The team of operators was composed of three dentists, two dental hygienists and four dental students from San Raffaele Hospital, Vita-Salute San Raffaele University. The team visited all the children in the School. In each class, operators initially gave a twenty-minute lesson to the children about the importance of oral health, nutrition and proper oral hygiene. Then, once parental consent was obtained in written form, children were visited by the dentists. After careful intra-oral examination, the children were also interviewed and asked some specific questions with the aid of a short questionnaire (Table A1). The questionnaire tapped into children’s attitudes towards home oral hygiene, and also provided some additional clinical anamnestic information.

The detection of DDE was based on a careful clinical observation of the following clinical aspects: reduced enamel thickness consisting of shallow or deep fossae with horizontal or vertical grooves (hypoplasia) and anomalies in tissue translucency consisting of white or yellowish/brownish areas (diffuse opacities and demarcated opacities) [2]. Three surfaces were examined: buccal, occlusal/incisal and lingual/palatal of all permanent teeth. Other diagnostic criteria were also considered: (a) A tooth was considered present when any portion of the crown had erupted through the mucosa; (b) When an enamel defect was present in the erupted part, it was recorded; (c) In case of doubt with respect to the presence of an abnormality, the dental surface was classified as “normal”; and (d) A surface with a single abnormality, less than 1 mm in diameter, was also classified as “normal” [2].

The following variables were then collected in the whole sample.

(A) Indices of oral health and prevention:
  - Number of decayed teeth;
  - Number of filled teeth;
  - Decayed, filled and missing teeth (DMFT) index;
  - Fissures sealants (on permanent and deciduous teeth);
  - Topical fluoride treatments at the dentist (anamnestic data);
  - Recurrent aphthous stomatitis (RAS).
(B) Children’s attitudes toward home oral hygiene:
  - Psychosocial attitudes towards home oral hygiene were assessed via a short questionnaire with multiple-choice format, and scored according to the coding system specified below, at the end of this paragraph (see also Appendix A).

All data were assessed with the explicit agreement of all operators, both in the case of clinical observations and for participants’ questionnaire answers. More specifically, the clinical observation involved more than a single operator, and the case ascertainment was established with the agreement of all the involved operators. Similarly, the answers to the questionnaire were obtained, and thus recorded, by interviewing each single child through a questionnaire-based interview. The operators gave this questionnaire full importance.

To reduce possible sources of bias among operators, the team underwent a training period of one week, held before the beginning of the project. During the training period, the operators focused on how to administer the questionnaire, and how to coordinate clinical examination on a total of 50 children visited at the Dental Clinic of San Raffaele Hospital with the questionnaire interview. Each data-set obtained by the young patients was completed with the agreement of all operators, and each case was discussed when unclear. In addition. the questionnaire-based interviews were always conducted by 2–3 operators, working simultaneously. Groups of operators were often switched and interchanged.

All the recorded data were handled at a patient level. Data about fissure sealants were treated as a dichotomous variable (i.e., by considering the absence vs. presence of the recorded condition). Data about decayed/filled teeth (and the DMFT index) were handled always at a patient level, by grouping together participants with a number of decayed/filled teeth ≥1. Children’s attitudes towards home oral hygiene were assessed via a short questionnaire. Each answer was coded by adopting a categorical multiple-choice answering format for each of the five questions entailed in the questionnaire. More specifically, a score was assigned to each answer. The scores were then summed up, for each child, to form a total score, which could range from 0 to 10. On the basis of their total scores, children were thus assigned to one of three mutually exclusive categories: (a) “poor attitudes towards of home oral hygiene” (scores 0–4); (b) “mixed/intermediate attitudes towards home oral hygiene” (scores 5–7); and (c) “highly positive attitudes towards home oral hygiene” (scores 8–10). This procedure led to the formation of an “attitude towards home oral hygiene” (AOH) index to be used in the analyses, resulting in three levels expressed as Low, Medium, High.

An a-priori sample size analysis was performed on the basis of preliminary data of the study, in order to achieve a minimum of 80% power with alpha 0.05 for the primary outcome (decayed teeth), which resulted in a minimum required sample size of 389 subjects.

### Statistical Analyses

All statistical analyses were performed using the SPSS software v. 21 (IBM, Armonk, NY, USA). The independence vs. association between categorical variables was assessed through chi-squared tests, complemented by odds ratios (ORs) computations. With respect to the AOH index (which consisted of three mutually exclusive categories: poor vs. moderate vs. highly positive attitudes towards home oral hygiene), the analysis focused on the (two-tailed) probability of obtaining a certain observed distribution of values in a 2 × 3 contingency table (nonparametric testing) [13], compared with the expected values for each of the 2 × 3 = 6 cells of the design. Odds ratios (ORs), 95% confidence intervals (CIs) and Z-tests were computed to further illuminate, in terms of risk factors, the observed significant associations between clinical and/or psychological variables. The threshold for statistical significance was set at *p* = 0.05 for all statistical tests.

## 3. Results

**Figure 1:**
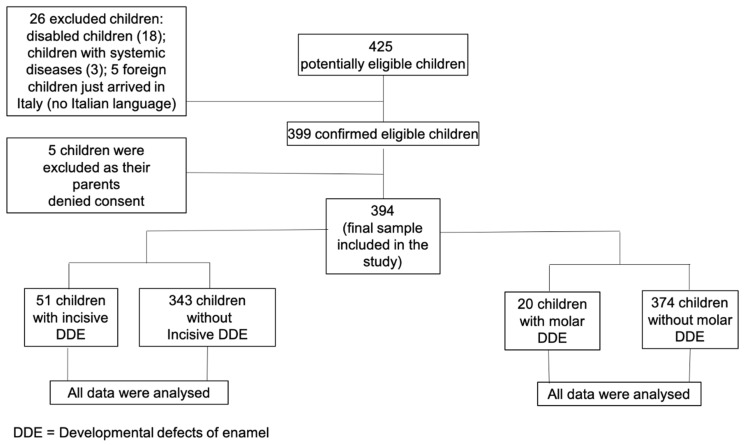
Study flow chart.

Among a set of *n* = 425 potentially eligible students from the School Sabin, Milan, *n* = 399 children were confirmed as eligible participants for the study. The flow chart of the study is reported in Figure 1. All 394 children obtained parental consent to receive a dental visit and were thus included in the analysis. All the data from the 394 children were analyzed, as there were not any missing or inconsistent data. In the case of temporary incomplete data, enrolled children were re-contacted to provide the missing information and complete the form. Thus, the statistical analyses were calculated on a whole final sample of *n* = 394 participants. Table 1 reports the sample’s main demographics (age and gender).

Our sample of children was not exposed to confounding factors such as, for instance, fluorine in the public waters of the city or specific oral health prevention programs conducted in the community of reference.

The numbers of participants assigned to each exposure category are reported in Table 2. This information is complemented by the results of chi-squared tests of independence (with Yates’ continuity corrections if intra-cell expected n.s. < 5).

Among the 394 subjects (mean age 8.9 ± 1.4), 5% (females) and 6% (males) of participants, respectively, had at least one molar affected by DDE and 12% (females) and 14% (males) of participants had at least one incisor affected by DDE (Table 1).

In general, no significant associations were observed for any of the examined clinical categories (see Table 2). The AOH index showed that most participants with molar DDE manifested either a poor (40.9%) or substantially positive (36.4%) attitude towards home oral hygiene, whereas only a few showed an attitude in between these two categories (22.7%). As shown in Table 2, a strong relationship was found between the presence of molar DDE and children’s positive vs. negative psychosocial attitudes towards home oral hygiene (for children with positive attitude: OR = 2.53, 95% CI (1.39–4.59)); Z-test = 3.052, *p* = 0.002; for children with negative attitude: OR = 1.32; 95% Cl: 0.78–2.24; *z* = 1.046 *p* = 0.295). The probability of finding DDE in our sample of examined children was approximately more than doubled (i.e., 2.53 times higher) for children with positive psychosocial attitudes towards home oral hygiene, and about 1.32 times higher for children with a negative attitude towards home oral hygiene.

Finally, given the specific nature of our research design—and given our declared interest into primary clinical and psychological outcomes only—no further statistical subgroup- or interaction-analysis was performed.

## 4. Discussion

This is, to our knowledge, the first study investigating the prevalence of DDE in a sample of North Italian children with mixed dentition, its possible associations with general oral health and preventive dentistry, and—importantly—with childrens’ declared psychosocial attitudes toward home oral hygiene.

In the evaluated sample of 394 children with mixed dentition (mean age: 8.9 ± 1.4), a prevalence of 5% and 6% (observed among females and males, respectively) was recorded for at least one molar affected by DDE and a prevalence of 12% and 14% (among males and females, respectively), for at least one incisor affected by DDE.

No significant associations were observed with any of the examined clinical categories such as: presence of caries or of other clinical oral health indices, presence of decayed or filled teeth, DMFT ≥ 1; presence of sealants; professional applications of fluoride substances; presence of recurrent aphthae. However, a remarkable association was found between the presence of molar DDE and children’s attitudes towards home oral hygiene. As a result, the probability of finding DDE in our examined sample was more than doubled for children with more positive psychosocial attitudes towards home oral hygiene, than for children with medium level attitudes towards home oral hygiene.

This sample was composed of participants with mixed dentition. Thus, the data on the prevalence of DDE can be considered reliable and generalizable for the population of Italian children with mixed dentition, not systematically exposed to risk factors for DDE. These data are, of course, not comparable with those of studies that evaluated the prevalence of DDE in primary or permanent dentition. The present results also point to generally less deteriorated figures, if compared with those stemming from published data of participants living in somewhat more “risky” regions such as, for instance, those pertaining to a sample of 270 children (mean age: 9.9 ± 2.6) from West Poland (in which the observed prevalence was 25%) [19] or with data based on other “risky” groups, such as those reported in a study by Cruvinel et al., 2012, showing a prevalence of 72.5% in a sample of Brazilian pre-term children of age 6–7 years [20].

Furthermore, some differences in the prevalence of DDE can also be observed in studies in which children were visited on a dental chair, exposed to artificial light after prophylaxis and with the dental surfaces dried with air streams [20]. In general, for studies that set the evaluation of psychological states as their primary objective, it is better to detect DDE at school with the naked eye, to merely identify those DDE that are genuinely recognizable. In fact, in the dental chair DDEs that would not appear in normal light can be more easily identified. Since the present research aimed at detecting the predicted association between DDE and a psychological factor such as children’s attitude towards home oral hygiene, it was deemed more appropriate, in our case, to confine the ascertainment of DDE to a clinical examination performed without artificial light. Moreover, if the patient can easily recognize his/her DDE lesions, such an altered physical appearance could easily affect his or her (positive vs. negative) global attitude towards home oral hygiene [21] and also threaten his or her general well-being and positive social functioning [22].

In the present sample, the general oral health status of children affected by DDE can be considered “acceptable” or “good”, as 77% of the children with molar DDE (17 children out of 22) and 84.4% of children with incisive DDE (43 children out of 51), showed a DMFT-index = 0 (“excellent oral status”), which is quite close to the percentage of DMFT = 0 (i.e., 75.3–76.4%) observed in children without DDE. The quality of children’s oral health status assessed in this study appears to be slightly better, if compared to an analogous condition observed in a sample of 775 Brazilian children (8–12 years) with DDE [21], in which 65% (i.e., 504 children out of 775) showed a DMFT = 0 (338/533 children (63.4%) with incisor DDE and 159/247 children with molar DDE (64.3%)). In that sample, the researchers observed that the experience of dental caries was more common among children with enamel hypoplasia in their posterior teeth (reported OR = 2.79; 95% CI (1.05–6.51)) than among children without enamel hypoplasia. In our sample, by contrast, we did not observe any significant correlation between the presence of DDE and dental caries or the DMFT. The comparatively superior oral status observed in our sample could be due to the fact that we included only children with mixed dentition, while the Brazilian study [21] included only children up to 12 years with complete permanent dentition, thus a higher number of caries is justifiable. The Brazilian study [21] also evaluated the DDE on the dental chair, which could have increased the number of reported “cases.” On the other hand, the data on the prevalence of dental caries among children are encouraging, given the stated goal of the World Health Organization to achieve, by 2020, a threshold of 50% percent with 5–6-year-olds children caries free and an average of no more than 3 DMF teeth, at 12 years of age [23].

In the present research, the attention given to children’s oral health by their parents was indicated by the request of preventive measures, such as professional fluoride treatments at the dental office and/or sealants in permanent or deciduous teeth. In general, these indicators were comparable in children with or without DDE: 15.6% and 13.6% of children, respectively with incisive or molar DDE, received professional fluoride treatments compared to 11.4% and 11.8% of children without DDE; while 19.6% and 18.2% of children with DDE received sealants of permanent teeth compared to 13.9% and 14.5% in children without. These observations indicate that children with DDE are often healthy children, submitted to primary prevention procedures (professional fluoride application and sealants of permanent teeth). These observations converge with those from Vergas-Ferreira et al., 2014 [21]. In their study, these authors discussed the commencement of brushing with fluoride before the age of 3 as the only indicator to assess attention given to oral health, and also observed that children with DDE tended to start earlier (and more frequent) fluoride brushing, if compared to children without (ORs = 1.62 for incisive DDE and 1.32 for molar DDE) [16]. However, it should be noted that the use of a single specific type of toothpaste is often associated with both financial and taste choices. The three indicators employed in the present study, by contrast, should go beyond these limits and would enable a more reliable assessment of parents’ attention to the oral condition of their children with DDE. Based on the results of the present study, DDE alone does not seem to influence parents’ behaviors concerning their children’s oral health preservation, even though common complications tied to enamel defects include tooth sensitivity and occlusal dysfunction [24].

A remarkable finding in the present study was the strong association between DDE and children’s psychological attitudes towards home oral hygiene. Most children with molar DDE showed either a relatively negative (40.9%) or a relatively positive (36.4%) attitude towards home oral hygiene (as reflected in the AOH index). These data are in contrast with those of children without DDE, who fell more frequently in the middle category. his suggests that, in general, children with molar DDE would need to be more intensely motivated by the dental hygienist and/or their parents to daily oral hygiene; or, in other cases, on the contrary, that children with molar DDE pay particular attention to their oral health. In this respect, it must be recognized that both motivational and cognitive factors play a pivotal role in human relations, which in turn are driven by—and have implications for—people’s perceptions, decisions and behaviors both at the individual and group relational level [25,26,27,28,29,30].

From a clinical point of view, these results suggest that molar DDE could play a role in the motivation towards home oral hygiene. The inesthetic appearance of anterior teeth does not always contribute to strengthen a positive attitude towards home oral hygiene while the molar enamel defect seems to have this effect. There is a potential for molar DDE to exert negative effects because this type of enamel defect is more severe, and potentially more dangerous for oral health (e.g., for caries, dentin sensitivity, occlusal and masticatory problems). This effect should be stronger for molar than incisive DDE, which are often only associated with a negative esthetics. This is an interesting point, considering that the restorative procedures for the altered enamel structure in molar DDE often create difficulties with anesthetizing the tooth and the bonding of the restoration to the enamel, such that the general clinical approach to DDE should be—once more—prevention [31]. Therefore, it would seem that clinicians should definitely foster the formation and maintenance of positive attitude towards oral hygiene at home, especially for children affected by molar DDE. This could be encouraged by proper school based oral health programs that seem to be able to bring a significant improvement in the oral health level of preschool children, acting on their related psychosocial attitudes and their parents knowledge [11,14,15], in agreement with the current guidelines concerning DDE, which direct clinicians to implement preventive rather than conservative/restorative procedures.

### Main Limitations of the Study

One limitation of the present study is that dental visits were performed at school, i.e., without the dental chair equipment available to the operators. However, as stated above, we were also aware that the naked eye observation could have been particularly appropriate for the analysis of the associations of our clinical variables of interest with psychological/attitudinal factors. Nevertheless, some inaccurate data may have been resulted from administering questionnaires to children who may not have given precise answers. Despite these potentially adverse conditions, the reported strong association between molar DDE and the (positive vs. negative) attitudes towards home oral hygiene clearly emerged in our sample.

## 5. Conclusions

A prevalence of 5–6% for molar DDE and 12–14% for incisor DDE was detected in the area of Milan, North Italy.

A strong systematic relationship was found between the presence of molar DDE and children’s positive vs. negative psychosocial attitudes towards home oral hygiene. As most children with molar DDE showed either a negative (40.9%) or positive (36.4%) attitude towards home oral hygiene while children without DDE fell more frequently in the middle category, it seems that molar DDE could have an influence in children’s psychosocial attitudes towards home oral hygiene.

No other significant associations were observed for any of the examined clinical categories, such as the presence of caries or of other clinical oral health indices.

## Figures and Tables

**Table 1:** Age and gender distribution.

Variable	Incisor DDE (*n* = 51)	Molar DDE (*n* = 20)	Whole Sample (*n* = 394)
Mean Age	8.9 ± 1.4	8.9 ± 1.4	8.9 ± 1.4
Male *n* (%)	24 (12%)	12 (6%)	197 (100%)
Female *n* (%)	27 (14%)	10 (5%)	197 (100%)

DDE = Developmental defects of enamel.

**Table 2:** Report of number of children (and percentage among cases and controls) for each exposure category with significant associations with developmental enamel defects (DDE).

Exposure Factors	Categories	Incisor DDE			Molar DDE		
		Number (%) of Subjects without Incisor DDE	Number (%) of Subjects with Incisor DDE	Sig.	Number (%) of Subjects without Molar DDE	Number (%) of Subjects with Molar DDE Cases	Sig.
Decayed Teeth	No	272 (79.3%)	46 (90.2%)	0.06 n.s.	300 (80.6%)	18 (81.8%)	0.89 n.s.
	Yes	71 (20.7%)	5 (9.8%)		72 (19.4%)	4 (18.2%)	
Recurrent Aphthous Stomatitis	No	307 (89.5%)	43 (84.4%)	0.27 n.s.	332 (89.3%)	18 (81.8%)	0.28 n.s.
	Yes	36 (10.5%)	8 (15.6%)		40 (10.7%)	4 (18.2%)	
Filled Teeth	No	325 (94.7%)	51 (100%)	0.09 n.s.	355 (95.5%)	21 (95.5%)	0.99 n.s.
	Yes	18 (5.3%)	0		17 (4.5%)	1 (4.5%)	
DMFT	DMFT = 0	258 (75.3%)	43 (84.4%)	0.15 n.s.	284 (76.4%)	17 (77.3%)	0.92 n.s.
	DMFT ≥ 1	85 (24.7%)	8 (15.6%)		88 (23.6%)	5 (22.7%)	
Sealing of Deciduous Teeth	No	332 (96.7%)	51 (100%)	0.19 n.s.	361 (97.1%)	22 (100%)	0.41 n.s.
	Yes	11(3.3%)	0		11 (2.9%)	0	
Sealing of Permanent Teeth	No	295 (86.1%)	41(80.4%)	0.29 n.s.	318 (85.5%)	18 (81.8%)	0.63 n.s.
	Yes	48 (13.9%)	10 (19.6%)		54 (14.5%)	4 (1.8.2%)	
Topical Fluoride Treatments	No	304 (88.6%)	43 (84.4%)	0.37 n.s.	328 (88.2%)	19 (86.4%)	0.79 n.s.
	Yes	39 (11.4%)	8 (15.6%)		44 (11.8%)	3 (13.6%)	
AOH	Low	108 (31.4%)	16 (31.3%)	0.99 n.s.	115 (30.9%)	9 (40.9%)	0.005 **
					OR (odds ratio) for low AOH value = 1.32 (95% Cl: 0.78–2.24) *z* = 1.046 *p* = 0.295		
	Medium	179 (52.1%)	27 (52.9%)		201 (54%)	5 (22.7%)	
	High	56 (16.3%)	8 (15.6%)		56 (15.1%)	8 (36.4%)	
					OR (odds ratio) for high OHA value = 2.53 (95% Cl: 1.39–4.59) *z* = 3.052 *p* = 0.002		

** *p* < 0.01; n.s. = not significant; Sig. = Statistical significance; DMFT = Decayed Missing Filled Teeth index; AOH = Attitude towards home Oral Hygiene index; DDE = Developmental Defects of Enamel.

## Appendix A

**Table A1:** Questionnaire on home oral hygiene attitude (AOH index) and scores assigned to the answers.

	Questions	Answers	Scores 0–1–2
1	Do you like to brush your teeth?	Yes	2
		No	0
		Yes, but I get a little bored	1
		No, but I know it has to be done	1
		No, I do not, and I do not brush them.	0
2	How many times do you brush your teeth in a day?	1	0
		2	1
		3	2
		After each meal	2
		Never	0
3	When you’re at school or away from home do you brush your teeth too?	Yes	2
		No	0
		Not always	1
4	When you finish brushing your teeth, does someone check if you have done it correctly?	Yes	2
		No	0
		Sometimes	1
5	Do you like to feel that, after brushing, your mouth is fresh, and your teeth are clean?	Yes	2
		No	1
		I like it very much	2
		No, it bothers me	0
